# Clinical and functional significance of tumor/stromal ATR expression in breast cancer patients

**DOI:** 10.1186/s13058-020-01289-4

**Published:** 2020-05-15

**Authors:** Mysoon M. Al-Ansari, Maher Al-Saif, Maria Arafah, Abdelmonneim M. Eldali, Asma Tulbah, Taher Al-Tweigeri, Abdelhabib Semlali, Khalid S. Khabar, Abdelilah Aboussekhra

**Affiliations:** 1 Department of Molecular Oncology, King Faisal Specialist Hospital and Research Center, MBC#03, Riyadh, 11211 Saudi Arabia; 2 Department of Microbiology, Faculty of Science and Medical Studies, King Saud University, Riyadh, Saudi Arabia; 3 Molecular BioMedicine Program, Research Centre, King Faisal Specialist Hospital and Research Centre, Riyadh, 11211 Saudi Arabia; 4 Department of Pathology, King Saud University, PO BOX 2925, Riyadh, 11461 Saudi Arabia; 5 Department of Biostatistics, Epidemiology and Scientific computing, King Faisal Specialist Hospital and Research Center, Riyadh, 11211 Saudi Arabia; 6 Department of Pathology, King Faisal Specialist Hospital and Research Center, Riyadh, 11211 Saudi Arabia; 7 Department of Oncology, King Faisal Specialist Hospital and Research Center, Riyadh, 11211 Saudi Arabia; 8 Groupe de Recherche en Écologie Buccale, Faculté de Médecine Dentaire, Université Laval Québec, Local 1758, 2420 rue de la terrasse, Québec, G1V 0A6 Canada

**Keywords:** ATR, AUF-1, Breast cancer, Cancer-associated fibroblasts, Disease-free survival

## Abstract

**Background:**

Most breast cancer-associated fibroblasts (CAFs) are active and important cancer-promoting cells, with significant impact on patient prognosis. Therefore, we investigated here the role of the protein kinase ATR in breast stromal fibroblasts in the prognosis of locally advanced breast cancer patients.

**Methods:**

We have used immunohistochemistry to assess the level of ATR in breast cancer tissues and their adjacent normal tissues. Immunoblotting as well as quantitative RT-PCR were utilized to show the role of breast cancer cells and IL-6 as well as AUF-1 in downregulating ATR in breast stromal fibroblasts. Engineered human breast tissue model was also used to show that ATR-deficient breast stromal fibroblasts enhance the growth of breast cancer cells.

**Results:**

We have shown that the protein kinase ATR is downregulated in cancer cells and their neighboring CAFs in breast cancer tissues as compared to their respective adjacent normal tissues. The implication of cancer cells in ATR knockdown in CAFs has been proven in vitro by showing that breast cancer cells downregulate ATR in breast fibroblasts in an IL-6/STAT3-dependent manner and via AUF-1. In another cohort of 103 tumors from locally advanced breast cancer patients, we have shown that absence or reduced ATR expression in tumoral cells and their adjacent stromal fibroblasts is correlated with poor overall survival as well as disease-free survival. Furthermore, ATR expression in CAFs was inversely correlated with tumor recurrence and progression.

**Conclusion:**

ATR downregulation in breast CAFs is frequent, procarcinogenic, and correlated with poor patient survival.

## Background

While preoperative or neoadjuvant chemotherapy is the mainstay therapeutic strategy for locally advanced breast cancer tumors, it allows disappearance of the tumor (pathological complete response: PCR) in only 20–30% of breast cancer cases [1]. This prompted several researchers to search for genes and gene signatures that can predict the response to neoadjuvant therapy of breast cancer. These tumors are heterogeneous and composed of different types of cells, including fibroblasts. Cancer-associated fibroblasts (CAFs), which are the most active stromal cells, play major roles in the development and spread of breast cancer. Although these cells are not cancerous, they exhibit several features of cancer cells, such as high expression level of oncogenes, reduced level of tumor suppressor genes, and high proliferative and invasive capacities [2, 3]. Furthermore, several lines of evidence showed the presence of clear correlation between CAF-related gene signature and clinical outcome of breast cancer patients. These findings suggested an important role of stromal biology in tumor behavior and the consequent patient response to therapy [4].

DNA damage and replication stress are major sources of genomic instability and carcinogenesis [5]. The *ataxia telangiectasia* and Rad3-related protein (ATR) is a key protein kinase, which plays major roles in the cellular responses to these genotoxic stresses [6]. Indeed, ATR induces various genes and processes that allow cells to cope specifically with the different stresses and insults, in a timely manner. ATR induces cell cycle delay and promotes various DNA repair processes [7]. Furthermore, we have recently shown that ATR level is lower in cancer-associated fibroblasts as compared to their corresponding tumor counterpart fibroblasts (TCFs), and ATR inhibits the procarcinogenic effects of CAFs in a p53-dependent manner [8].

In the present study, we addressed the causes and consequences of ATR downregulation in breast stromal fibroblasts and the correlation between the ATR level and the clinical outcome of breast cancer patients. We have shown AUF1-dependent downregulation of ATR in CAF cells, and poor prognosis of patients bearing tumors expressing low level of ATR in both cancer cells as well as cancer-associated fibroblasts.

## Materials and methods

### Cells and cell culture

Breast fibroblast cells were obtained and used as previously described [9]. MDA-MB-231 and MCF-10A cells were purchased in 2011 from ATCC and were authenticated using short tandem repeat profiling by ATCC, propagated, expanded, and frozen immediately into numerous aliquots after arrival. The revived cells were utilized within 10 to 12 passages and not exceeding a period of 3 months and were cultured following the instructions of the company. Cells were regularly screened for mycoplasma contamination using MycoAlert Mycoplasma Detection Kits (Lonza). All supplements were obtained from Sigma (Saint Louis, MO, USA) except for antibiotic and antimycotic solutions, which were obtained from Gibco (Grand Island, NY, USA). Cells were maintained at 37 °C in a humidified incubator with 5% CO_2_.

Human IL-6 recombinant protein (hBA-184) (Santa Cruz, CA).

### Cellular lysate preparation and immunoblotting

This has been performed as previously described [10]. Antibodies directed against AUF1 (ab50692) and ATR (ab54793) were purchased from Abcam (Cambridge, MA), STAT3 and pSTAT3-Tyr705 (D3A7) from Cell Signaling (Danvers, MA), and glyceraldehydes-3-phosphate dehydrogenase (GAPDH, FL-335) from Santa Cruz (Santa Cruz, CA).

### RNA purification and qRT-PCR

Total RNA was purified using the TRI reagent (Sigma) according to the manufacturer’s instructions and was treated with RNase-free DNase before cDNA synthesis using the RT-PCR Kit (Clontech, USA). cDNA was amplified using the Platinum® *Taq* DNA Polymerase (Invitrogen). The RT^2^ Real-Time™ SYBR Green qPCR mastermix (Roche, Germany) was used, and the amplifications were performed utilizing the light cycler 480 (Roche, Germany). The melting-curve data were collected to check PCR specificity, and the amount of PCR products was measured by threshold cycle (Ct) values and the relative ratio of specific genes to *GAPDH* for each sample was then calculated. The respective primers are:

- *GAPDH*: 5′-GAGTCCACTGGCGTCTTC-3′ and 5′-GGGGTGCTAAGCAGTTGGT-3′.
- *ATR*: 5′-GTCATATACACTCCCTTTTCTTTA-3′ and 5′-GTCATATACACTCCCTTTTCTTTA-3′.
- *CDKN1A*: 5′-CAGAGGAGGCGCCAAGACAG-3′ and 5′-CCTGACGGCGGAAAACGC-3′.
- *CDKN2A*: 5′-CAACGCACCGAATAGTTACG-3′ and 5′-CAGCTCCTCAGCCAGGTC-3′.

### siRNA transfection

*IL-6*-siRNA, *STAT3*-siRNA, and control–siRNA were obtained from QIAGEN, USA. AUF1-siRNA (pSILENCER-AUF15), which targets all four AUF1 isoforms [11], was a generous gift from Dr. Gorospe. The transfections were carried out using the RNAi Fect reagent (QIAGEN) as recommended by the manufacturer.

### ATR-shRNA transfection

*ATR*-shRNA (KHD1318) expressed in sure silencing shRNA plasmid and the corresponding control plasmid were obtained from GenScript Corporation and were used to carry out transfection using human dermal fibroblast nucleofector 2000 transfection kit (Invitrogen, USA) following the protocol recommended by the manufacturer.

### Analysis of mRNA stability

Cells were challenged with actinomycin D (5 μg/ml) for various periods of time (0–6 h), and then total RNA was purified and assessed using qRT-PCR. One-phase exponential decay curve analysis (Sigma Plot) was used to assess the mRNA decay kinetics, considering the values at time 0 as 100%. The time corresponding to 50% remaining mRNA was considered as mRNA half-life.

### Conditioned media

Cells were cultured in medium without serum for 24 h, and then media were collected and briefly centrifuged. The resulting supernatants were used either immediately or were frozen at − 80 °C until needed.

### Cloning of ARE reporter constructs

The *ATR3* 3′UTRs were amplified from human fibroblast cDNA by RT-PCR using forward and reverse primers. The forward primer sequence is GGACTCCATATATGTGAAAT and the reverse primer is GTATTAAGAAAGCAGTTT. The primers were designed to contain (G/GATCC) BamHI and (T/CTAGA) XbaI overhangs. The PCR products were cloned in *BamH1* and *XBaI* sites in the 3′UTR of a post-transcriptional reporter comprising RPS30 ribosomal promoter and nanoluciferase as previously described [12, 13].

### Transfection and reporter activity measurements

Cells were plated at 4.10^5^ cells/ml per well in a 96-well plate and co-transfected with nanoluciferase post-transcriptional reporter containing the ATR 3′UTR sequences and control non-ARE 3′UTRs that was fused with firefly luciferase. The cells were transfected using Lipofectamine 2000 (Invitrogen) according to the manufacturer’s instructions. Transfections were performed in several replicates. After 16 h, cells were lysed and assayed for nanoluciferase activity using the dual Nano-Glo dual-luciferase assay kit (Promega) according to the manufacturer’s instructions and measured on a luciferase luminometer. Data were presented as mean ± SEM of normalized Nano-luciferase intensity/firefly intensity.

### Engineered human breast tissue (EHBT)

This has been performed as previously described [14, 15]. Briefly, the lamina propria was produced by mixing rat tail collagen type I (3 mg/mL) (Sigma, St. Louis, MO) with breast fibroblasts in fetal calf serum-supplemented culture medium for 4 days. This connective tissue was seeded with MDA-MB-231 to obtain the EHBT and was grown under submerged conditions until the total surface of the connective tissue is covered with epithelial cells. To produce a stratified epithelium, the EHBT was raised to an air-liquid interface for five more days to enable the epithelium to organize into its different strata including the basal layer.

### Immunohistochemistry staining on EHBT

Engineered human breast tissue was cut into 3-μm-thick sections, was mounted on saline-coated slides, and was incubated for 15 to 20 min in a hot air oven at 60 °C. Slides were then washed and were incubated for 32 min at 37°C with anti-Cyclin D1 and anti-Ki67 (diluted 1:200) and followed by an incubation with the secondary antibody ultraview universal HRP multimer as previously described [16]. The immunolocalized Ki67 and Cyclin D1 proteins were analyzed by an Olympus BX51 light microscope and DP72 Olympus digital camera (magnification × 200 and × 400) (Olympus America Inc., Center Valley, PA, USA).

### Patients and archived clinical materials

Formalin-fixed paraffin-embedded tissues were obtained from the Pathology Department at KFSH&RC with institutional review board approval (RAC#2160005). The study cohort consisted of 103 locally advanced breast cancer patients who were diagnosed between 2006 and 2013, with a median follow-up time of 52.6 months. Written informed consent was not required and a waiver was granted since samples were anonymized to the research team.

### Immunohistochemistry staining on FFPE tissues

Immunohistochemistry for ATR was done on formalin-fixed, paraffin-embedded tissue using anti-ATR from Abcam company (ab54793) overnight at a dilution of 1:500, and they were stained using automated staining platform (Ventana). Envision + polymer (ready to use; Dako) was used as a secondary antibody. Color was developed with 3,3′-diaminobenzidine (DAB), and instant hematoxylin (Shandon) was used for counterstaining. The ATR level was evaluated and verified by two qualified pathologists, who scored both the proportion of positive cells as well as the intensity of ATR expression in both cancer cells and their stromal fibroblasts.

### Statistical analysis

Statistical analysis was performed by the software package SAS version 9.4 (SAS Institute Inc., Cary, NC, USA). Continuous variables were compared by Student’s *t* test, and *P* values of 0.05 and less were considered as statistically significant. The Kaplan-Meyer method was used in survival tables and curves, and the different subgroups were compared by the log-rank test.

## Results

### ATR is downregulated in active breast cancer-associated fibroblasts

**Fig. 1:**
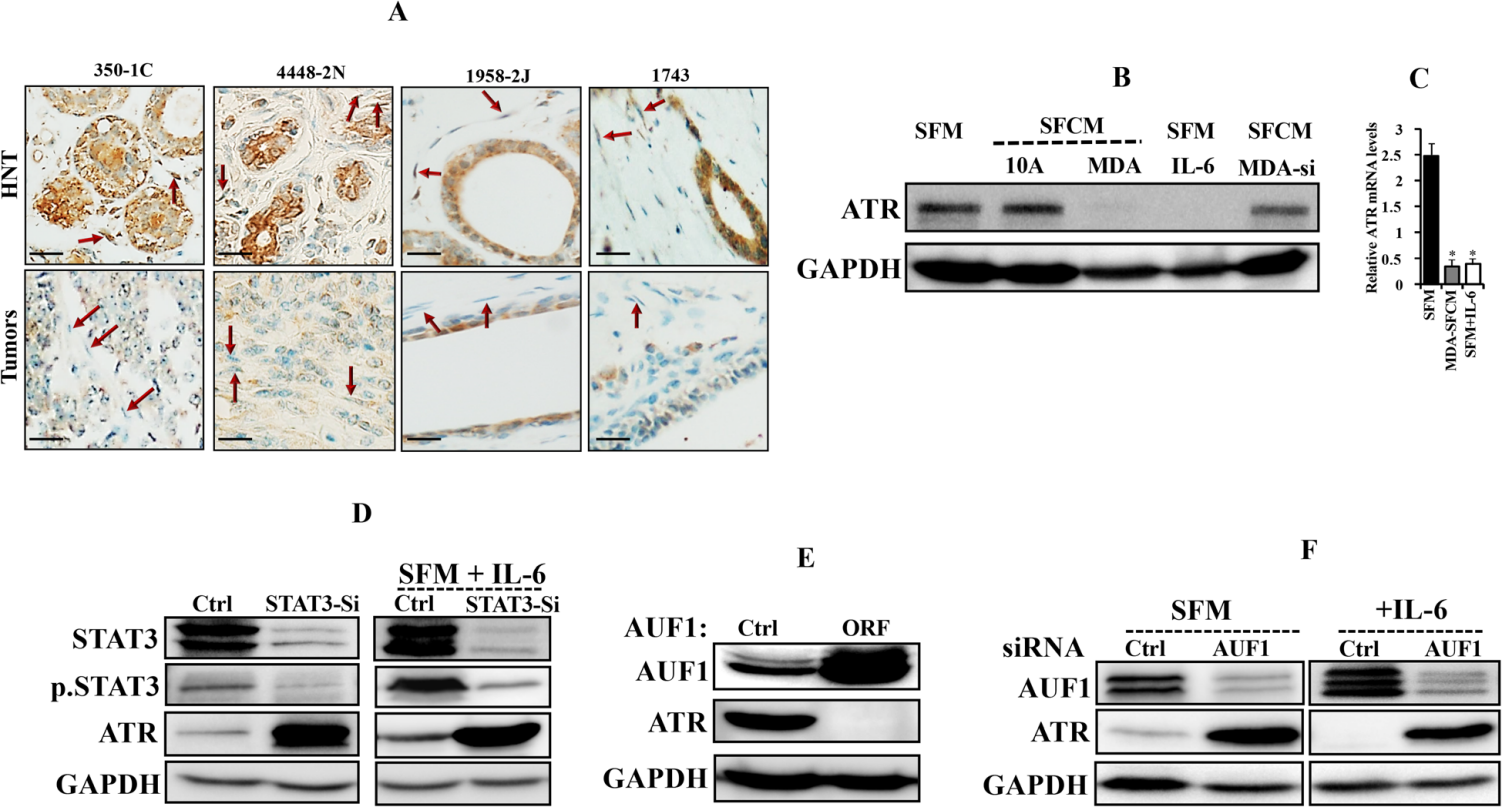
Breast cancer cells downregulate breast stromal fibroblast ATR in an IL-6/STAT3/AUF1-dependent manner. **a** FFPE tissues isolated from tumors as well as histologically normal tissues (HNT) were immunostained using anti-ATR antibody. Arrows indicate stromal fibroblasts. Scale bar = 50 μm. **b** TCF-64 cells were treated either with SFM (control) or with SFM containing IL-6 (3.5 mng/mL) or with SFCM obtained from MCF-10A (10A) and MDA-MB-231 (MDA). In addition, TCF-64 cells were exposed to SFCM obtained from MDA-MB-231 cells wherein IL-6 was knocked down using specific siRNA (MDA-si). Whole cell lysates were prepared and used for immunoblotting analysis. **c** TCF-64 cells were treated as indicated, and then total RNA was purified and used for qRT-PCR. Error bars represent mean ± SD for three different experiments and *P < 3.3.10^− 15^. **d** TCF-64 cells were transfected with STAT3-siRNA or a scrambled sequence (Ctrl), and then were treated as indicated and cell lysates were prepared and used for immunoblotting analysis using antibodies against the indicated proteins. GAPDH was used as internal control. **e** TCF-64 cells were transfected with either p37^AUF1ORF^ or an empty vector (Ctrl), and then cell lysates were prepared and used for immunoblotting analysis. **f** TCF-64 cells were transfected with AUF1 specific siRNA or with a scrambled sequence (Ctrl), and then both cells were treated as indicated. Cell lysates were prepared and used for immunoblotting analysis

We started the present study by assessing the ATR expression level in 10 paraffin-embedded sections from breast cancer tissues, including both tumor as well as their adjacent histologically normal tissues, by immunostaining using anti-ATR antibody. Figure 1a shows a sharp decrease in the ATR protein level in both fibroblasts and tumor cells present in tumors as compared to their counterparts present in normal adjacent tissues. This differential expression was obtained in 9 out of 10 pairs, while in the 10th pair, the ATR level was similarly low in both tumor and normal tissues. In 7 out of 10 cases, the level of ATR in stromal fibroblasts was matching the level of ATR in the corresponding cancer cells. This indicates that ATR is downregulated in cancer cells as well as in their neighboring CAFs in breast cancer tissues as compared to their respective adjacent normal tissues.

### Breast cancer cells downregulate breast stromal fibroblast ATR in an IL-6/STAT3/AUF1-dependent manner

We have recently shown that breast cancer cells activate breast stromal fibroblasts (BSFs) in an IL-6-dependent manner [17]. Therefore, we sought to investigate the possible implication of breast cancer cells in ATR downregulation in BSFs through IL-6 signaling. To this end, TCF-64 cells (derived from histologically normal breast cancer tissue) were cultured with serum-free medium (SFM) used as negative control, and serum-free conditioned media (SFCM) derived from both the non-carcinogenic epithelial cells MCF-10A (used as control) as well as the highly invasive breast cancer cells MDA-MB-231. Cells were harvested after 24 h, and whole cell lysates were prepared and used for immunoblotting analysis utilizing specific antibodies, and GAPDH was used as internal control. Figure 1b shows that while SFCM from MCF-10A cells had only marginal effect on the ATR level, SFCM from MDA-MB-231 cells abolished ATR expression as compared to the basal level (SFM). Interestingly, adding IL-6 (3.5 ng/mL) to SFM had an effect similar to that of SFCM from MDA-MB-231 cells (Fig. 1b). Similarly, SFCM from MDA-MB-231 cells and recombinant IL-6 potently reduced the *ATR* mRNA level (Fig. 1c). On the other hand, IL-6 knockdown by specific siRNA in MDA-MB-231 cells suppressed the paracrine suppressive effect of these cells on the expression of ATR in BSFs (Fig. 1b). This indicates that breast cancer cells downregulate ATR in BSFs in an IL-6-dependent manner. To show the possible implication of STAT3 in this process, we downregulated STAT3 by specific siRNA in TCF-64 cells, which strongly upregulated ATR and abolished IL-6-related ATR downregulation (Fig. 1d). Since IL-6-dependent ATR downregulation occurred at the mRNA level and AUF1 is a major post-transcriptional regulator and downstream effector of STAT3 in breast fibroblasts [17], we tested the effect of ectopic expression of p37^AUF1^ in TCF-64 cells on the expression of ATR. An empty vector was used as negative control. Figure 1e shows AUF1-dependent ATR downregulation. To further elucidate the role of AUF1 in ATR expression in response to IL-6 in BSFs, AUF1 was knocked down by specific siRNA (N64F1si) (a scrambled sequence was used as control) (N64Ctrl), and then cells were exposed either to SFM or SFM containing IL-6. Figure 1f shows that AUF1 downregulation strongly increased the ATR level and also inhibited the IL-6-related ATR downregulation. This shows that IL-6-related ATR inhibition in BSFs is AUF1-dependent and that AUF1 represses the expression of ATR.

### AUF1 destabilizes the *ATR* mRNA through binding its 3′UTR

**Fig. 2:**
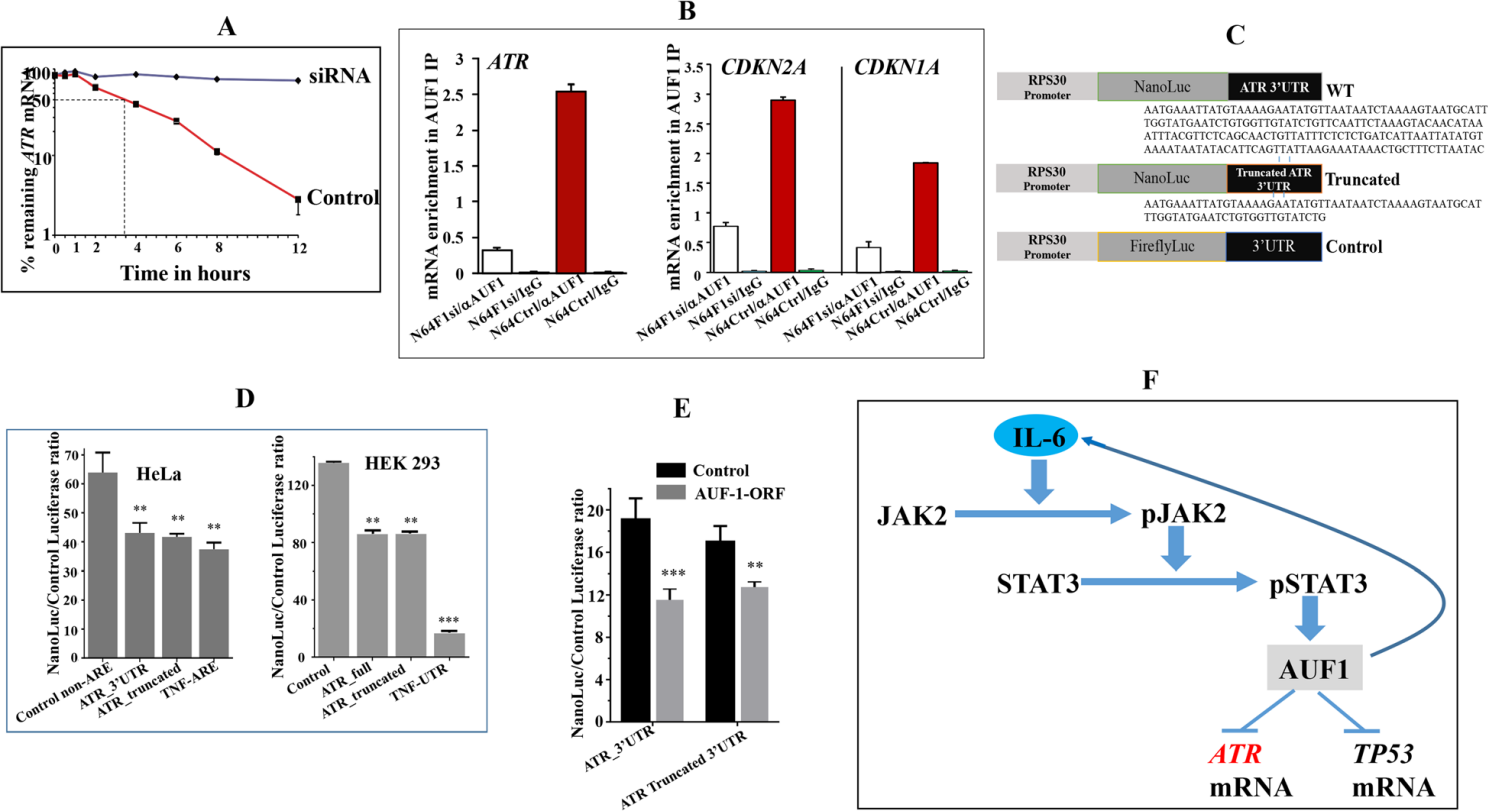
AUF1 destabilizes the *ATR* mRNA through binding its 3′UTR. **a** TCF-64 cells bearing either a scrambled sequence (control) or AUF1siRNA (siRNA) were treated with actinomycin D for the indicated periods of time. Total RNA was extracted and the remaining amounts of the *ATR* mRNA were assessed using qRT-PCR. The values at time 0 were considered as 100%. The dashed lines reveal the half-life (50% mRNA remaining). Error bars indicate mean ± SD (*n* = 3). **b** RNAs bound to the AUF1 protein were immunoprecipitated from the indicated cells using anti-AUF1 antibody or anti-IgG (used as control), and then the mRNAs of the indicated genes were amplified by qRT-PCR. Data were normalized to the levels of the highly abundant *GAPDH* mRNA in each IP sample and represented as the enrichment of each mRNA. Error bars indicate mean ± SD. **c** Scheme showing *ATR* 3′UTR constructs used in this study. **d** HeLa and HEK293 cells were co-transfected with the nanoluciferase (NanoLuc) reporters bearing the indicated constructs, and control firefly luciferase expression vector for 16 h. Cells were lysed and luciferase activity was quantitated as Nanoluc/Firefly luc intensity ratio. **e** CAF-64 cells expressing AUF1 ORF (p37^AUF1^) or not (control) were co-transfected with nanoluciferase (NanoLuc) reporters bearing the indicated 3′UTRs, and control firefly luciferase expression vector for 16 h. Cells were lysed and luciferase activity was quantitated as Nanoluc/Firefly luc intensity ratio. Data in **d** and **e** are mean ± SEM of triplicate readings of three experiments for each cell line (**P < 0.0005, ***P = 0.0001). **f** Schematic representation of IL-6-dependent downregulation of ATR through STAT3/AUF1-related destabilization of the *ATR* mRNA

To delineate the molecular link between AUF1 and ATR, we tested the possible implication of AUF1 in the turnover of the *ATR* transcript. Therefore, control and ATR-deficient cells were treated with actinomycin D for various periods of time, and then the proportion of the remaining *ATR* mRNA was assessed by quantitative RT-PCR (qRT-PCR). Figure 2a shows that the *ATR* mRNA half-life is 3 h 30 min in control cells and that AUF1 knockdown strongly stabilized the *ATR* transcript. This indicates that AUF1 plays a major role in the *ATR* mRNA turnover. To confirm this, we first investigated the possible binding of AUF1 to the *ATR* mRNA. To this end, whole cell lysates were prepared from N64F1si and N64F1Ctrl cells, and then AUF1-mRNA ribonucleoprotein complexes were obtained by IP using anti-AUF1 antibody (IgG was used for control cells) and were used for qRT-PCR. The immunoprecipitation with anti-AUF1 antibody yielded higher amplification of the *ATR*, *CDKN1A*, and *CDKN2A* mRNAs in control cells as compared to AUF1-deficient cells (N64F1si) (Fig. 2b). However, only marginal amplifications were obtained following immunoprecipitation with IgG (Fig. 2b). This shows that, like for the *CDKN2A* and *CDKN2B* mRNAs, AUF1 binds the *ATR* mRNA as well. To confirm the AUF1-related destabilization of the *ATR* mRNA and the implication of its 3′UTR in this interaction, the full-length *ATR* 3′UTR and artificially truncated form, along with control non-ARE 3′UTR, were subcloned into a post-transcriptional reporter [12, 13] (Fig. 2c). We found that full *ATR* 3′UTR caused significant reduction of the post-transcription reporter in both HEK293 and HeLa cell lines (Fig. 2d). Likewise, a truncated form (73-bases) of the *ATR* 3′UTR caused a similar reduction in both cell lines (Fig. 2d). As positive control, the strong *TNF* 3′UTR was used, and a highly significant effect was obtained (Fig. 2d). The effect of AUF1 on the full ATR 3′UTR and the truncated 3′UTR was examined in TCF-64 fibroblasts stably expressing p37^AUF1ORF^ or an empty vector used as control. There was statistically significant reduction (nearly 40%) of the post-transcription reporter with the full-length ATR and to a lesser degree with the truncated form (25% reduction) (Fig. 2e). Together, these results show that AUF1 binds the *ATR* 3′UTR and enhances the turnover of the corresponding message. This has been summarized in Fig. 2f.

### ATR-deficient breast stromal fibroblasts enhance the growth of breast cancer cells in engineered human breast tissue

**Fig. 3:**
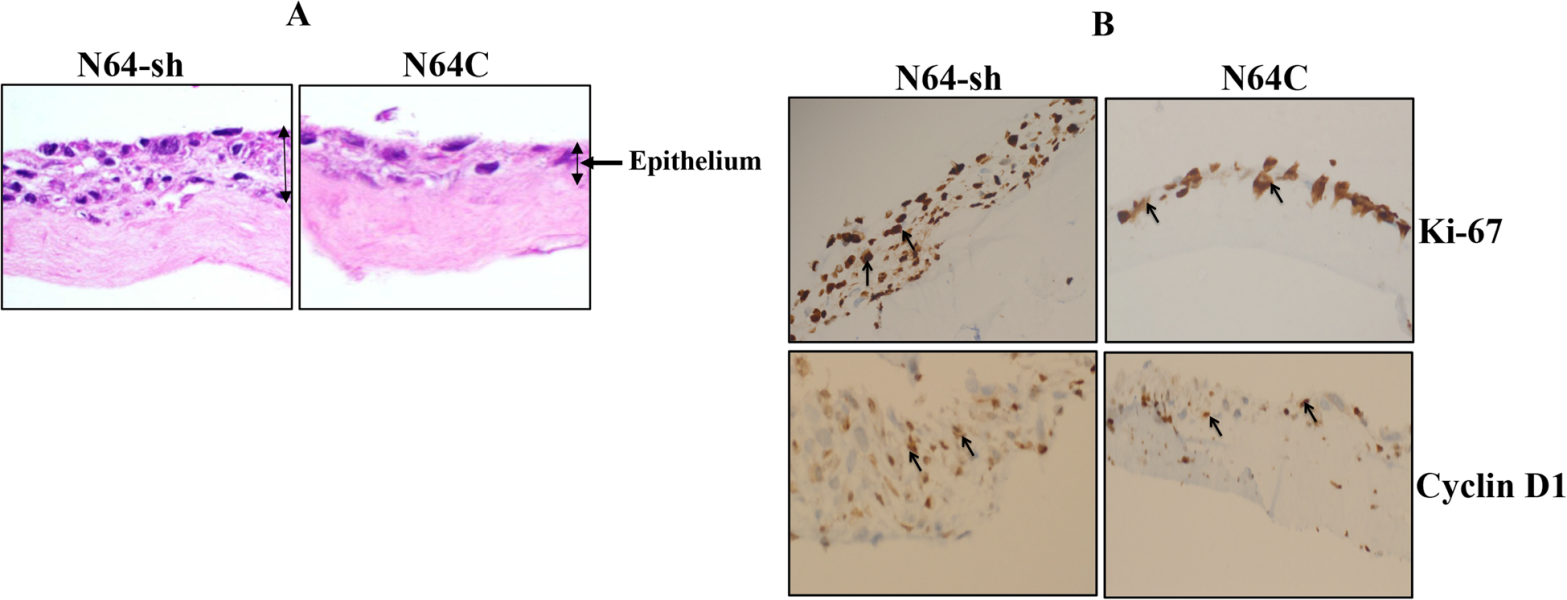
ATR-deficient breast stromal fibroblasts enhance the growth of breast cancer cells in engineered human breast tissue. Engineered human breast tissue was generated by seeding N64-sh or N64C cells with MDA-MB-231. **a** Histological features of EHBT (Envision × 40). **b** Immunohistochemistry staining on EHBT using antibodies against the indicated proteins

After showing the effects of ATR deficiency in 2D cultured cells, we decided to move our study closer to the in vivo setting by using an engineered human breast tissue (EHBT) model, as previously described by Semlali et al. [14, 15]. In this model, the connective tissue was formed with the TCF-64 cells expressing either specific ATR-shRNA (N64-sh, ATR-deficient) or a control vector (N64C) as previously shown [8], while the epithelium was generated with the highly aggressive and proliferative MDA-MB-231 cells. Figure 3a shows the generated EHBT structures, with striking differences in the organization and the width of the epithelium. Indeed, while the epithelium from the control tissue revealed a disorganized structure with a small epithelial layer, N64-sh cells generated a well-organized structure, with larger stratified multilayered epithelial tissue (Fig. 3a). This indicates more proliferative and aggressive breast cancer cells while adjacent to ATR-deficient stromal fibroblasts. This pro-proliferative effect of ATR-deficient cells was confirmed by immunostaining showing an increase in the staining of Ki-67 and cyclin D1 in the epithelium adjacent to N64-sh cells as compared to control (Fig. 3b).

### Correlation of ATR expression in cancer as well as stromal fibroblasts with clinicopathological parameters

Next, we sought to investigate the predictive value of ATR expression levels in cancer cells as well as in stromal fibroblasts as a candidate biomarker for clinical outcome of patients with LABC. The clinicopathological features of the enrolled patients (*n* = 103) are listed in Additional file 1. ER+/Her2+ patients (26) represented 25%, ER+/Her2− (32) represented 31%, and ER−/Her+ (24) represented 23%, while ER−/Her2− (21) represented 20%. Remarkably, 70% of the patients were less than 50 years old, and the same proportion had high tumor stage, while 52% of the tumors were of grade 3. Thirty-nine patients developed recurrence and 19 died (Additional file 1). Notably, 60% of patients had tumor sizes more than 5 cm (Additional file 1).

**Fig. 4:**
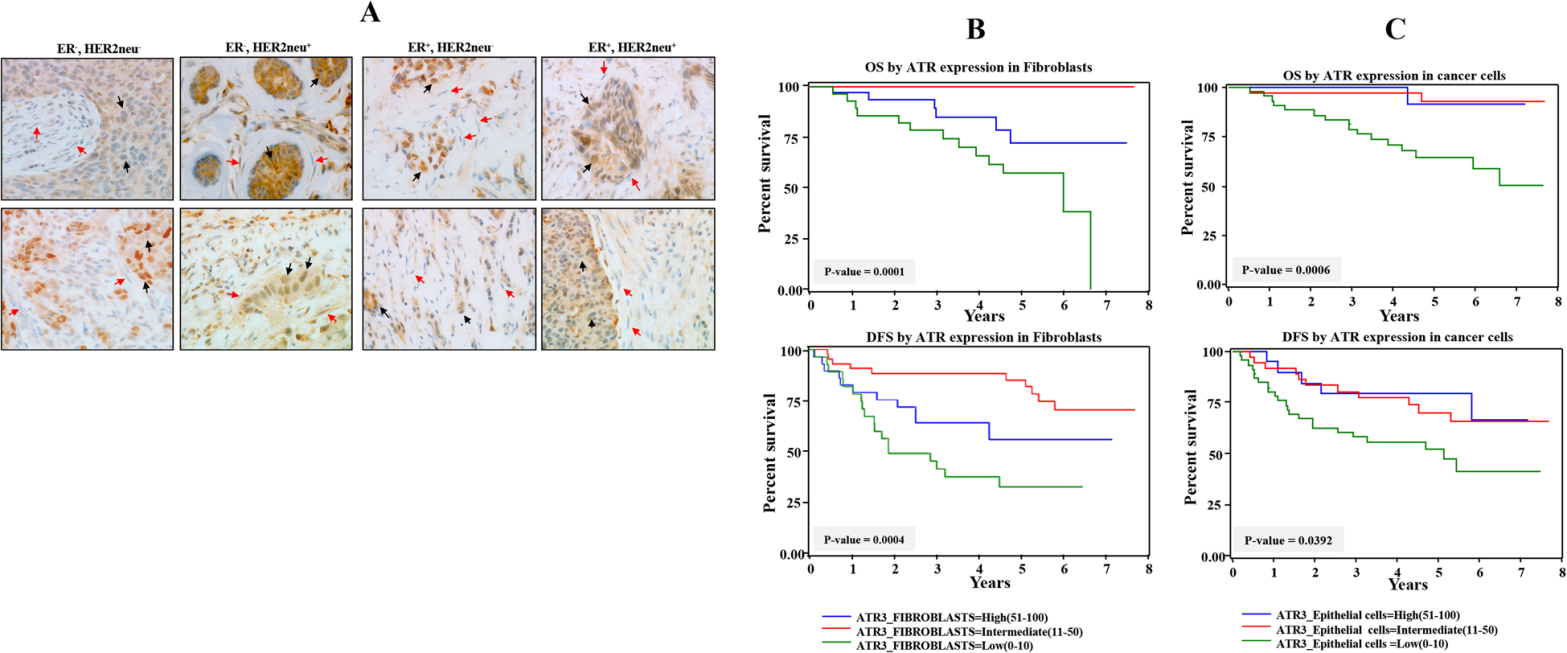
Loss of ATR in stromal and tumoral cells is predictive of poor disease-free and overall survival. **a** Tissue sections cut from formalin-fixed paraffin-embedded breast tumors (different subtypes) were immunostained with anti-ATR antibody. Red arrows indicate stromal fibroblasts, while black arrows indicate cancer cells. (Envision × 60). **b**, **c** Kaplan-Meier analysis of overall survival (OS) and disease-free survival (DFS)

A total of 103 breast pretreatment tumor tissues were assessed for ATR expression in both cancer as well as stromal cells. The reading of slides was performed and verified by 2 independent qualified pathologists. Figure 4a shows ATR immunostaining in breast cancer tissues in both epithelium and stroma. The ATR immunostaining in both types of cells was classified into three subgroups: low (0–10% ATR positive cells), intermediate (11–50% ATR positive cells), and high (51–100% ATR positive cells). Additional file 2 shows that the ATR expression level was low or completely lost in 46/103 fibroblast and 38/103 epithelial cells, intermediate in 30/103 fibroblast and 19/103 epithelial cells, and high in 27/103 fibroblast and 46/103 epithelial cells. The ATR expression in the different subtypes indicated association with ER/Her2. Indeed, significant correlation (*p* value = 0.0212) was observed between low ATR level in fibroblasts and lack of ER or Her2 (Additional file 2). However, no correlation was observed between ATR level in cancer cells and ER/Her2 expression levels (Additional file 2). Table 1 shows that the expression level of ATR in CAFs did not correlate with tumor stage and the Ki-67 index. However, ATR expression in CAFs was significantly associated with tumor grade and progression (*P* = 0.0473 and *P* = 0.0003, respectively) as well as patient survival (*P* < 0.0001), while it was inversely correlated with tumor recurrence (*P* = 0.0017) (Table 1). Patient survival was also highly correlated with the level of ATR in breast cancer cells (*P* = 0.0006) (Table 1).

**Table 1:** Correlations between ATR expression and clinicopathological characteristics in breast cancer patients

**Parameter**	**Total (*n*** **= 103) (%)**	**ATR in stromal fibroblasts**			***P*value**
		**High**	**Intermediate**	**Low**	
**Stage**					
T2	30 (29.13)	6 (5.83)	17 (16.50)	7 (6.80)	
T3	29 (28.16)	10 (9.71)	13 (12.62)	6 (5.83)	0.3308
T4	43 (41.75)	14 (13.59)	14 (13.59)	15 (14.56)	
Tx	1 (0.97)	0 (0.00)	1 (0.97)	0 (0.00)	
**KI-67 index**					
0	85 (84.16)	26 (24.75)	37 (36.63)	23 (22.77)	
≤ 15	2 (1.98)	0 (0.00)	2 (1.98)	0 (0.00)	0.7652
>15	14 (13.86)	5 (4.95)	5 (4.95)	4 (3.96)	
**HIS subtype**					
None invasive	1 (0.97)	1 (0.97)	0 (0.00)	0 (0.00)	
Invasive ductal Ca	85 (82.52)	23 (22.33)	40 (38.83)	22 (21.36)	
1,4	3 (2.91)	1 (0.97)	1 (0.97)	1 (0.97)	0.5887
Invasive ductal Ca with DCIS	11 (10.68)	3 (2.91)	4 (3.88)	4 (3.88)	
Infiltrating lobular Ca	1 (0.97)	0 (0.00)	0 (0.00)	1 (0.97)	
3, 4	1 (0.97)	1 (0.97)	0 (0.00)	0 (0.00)	
Other	1 (0.97)	1 (0.97)	0 (0.00)	0 (0.00)	
**Recurrence**					
No	63 (61.76)	18 (17.65)	35 (34.31)	10 (9.80)	0.0017
Yes	39 (38.24)	11 (10.78)	10 (9.80)	18 (17.65)	
**Grade**					
G1	3 (2.91)	1 (0.97)	1 (0.97)	1 (0.97)	
G2	46 (44.66)	9 (8.74)	27 (26.21)	10 (9.71)	0.0473
G3	54 (52.43)	20 (19.42)	17 (16.50)	17 (16.50)	
**Progression**					
**No**	73 (72.28)	13 (12.87)	40 (39.60)	20 (19.81)	0.0003
**Yes**	28 (27.72)	14 (13.86)	4 (3.96)	18 (9.9)	
**Survival status**					
Alive	84 (81.55)	24 (23.30)	45 (43.69)	15 (14.56)	< 0.0001
Dead	19 (18.45)	6 (5.83)	0 (0.00)	13 (12.62)	
**Duration of clinical follow-up (years, mean ± SD)**		4.16500000	5.00755556	3.87464286	0.0390
**Parameter**	**Total (*n*** **= 103) (%)**	**ATR in cancer cells**			***P*value**
		**High**	**Intermediate**	**Low**	
Tumor size					
≤ 5	35 (40.23)	7 (8.05)	15 (17.24)	13 (14.94)	0.4008
>5	52 (59.77)	8 (9.20)	17 (19.54)	27 (31.03)	
**Stage**					
T2	30 (29.13)	6 (5.83)	12 (11.65)	12 (11.65)	
T3	29 (28.16)	6 (5.83)	10 (9.71)	13 (12.62)	0.6772
T4	43 (41.75)	6 (5.83)	16 (15.53)	21 (20.39)	
Tx	1 (0.97)	1 (0.97)	0 (0.00)	0 (0.00)	
**KI-67 index**					
0	85 (84.16)	13 (12.87)	32 (31.68)	40 (39.60)	0.1525
≤ 15	2 (1.98)	0 (0.00)	1 (0.99)	1 (0.99)	
>15	14 (13.86)	6 (5.94)	3 (2.97)	5 (4.95)	
**HIS subtype**					
None invasive	1 (0.97)	0 (0.00)	1 (0.97)	0 (0.00)	
Invasive ductal Ca	85 (82.52)	13 (12.62)	33 (32.04)	39 (37.86)	
1,4	3 (2.91)	0 (0.00)	1 (0.97)	2 (1.94)	0.1336
Invasive ductal Ca with DCIS	11 (10.68)	4 3.88)	2 (1.94)	5 (4.85)	
Infiltrating lobular Ca	1 (0.97)	0 (0.00)	1 (0.97)	0 (0.00)	
3, 4	1 (0.97)	1 (0.97)	0 (0.00)	0 (0.00)	
Other	1 (0.97)	1 (0.97)	0 (0.00)	0 (0.00)	
**Recurrence**					
No	63 (61.76)	14 (13.73)	26 (25.49)	23 (22.55)	0.1000
Yes	39 (38.24)	5 (4.90)	11 (10.78)	23 (22.55)	
**Grade**					
G1	3 (2.91)	1 (0.97)	1 (0.97	1 (0.97)	
G2	46 (44.66)	10 (9.71)	14 (13.59)	22 (21.36)	0.6015
G3	54 (52.43)	8 (7.77)	23 (22.33)	23 (22.33)	
**Progression**					
**No**	73 (72.28)	17 (16.83)	29 (28.71)	27 (26.73)	0.0570
**Yes**	28 (27.72)	2 (1.98)	9 (8.91)	17 (16.83)	
**Survival status**					
Alive	84 (81.55)	18 (17.48)	36 (34.95)	30 (29.13)	0.0006
Dead	19 (18.45)	1 (0.97)	2 (1.94)	16 (15.53)	
**Duration of clinical follow-up (years)**		4.16500000	5.00755556	3.87464286	0.1883

### ATR expression in both breast cancer cells and their stromal fibroblasts predicts survival

Kaplan-Meier plots shown in Fig. 3b, c indicate significant association between ATR expression levels in both tumoral and stromal cells and patient’s overall survival (OS) as well as disease-free survival (DFS). Indeed, patients with low ATR expression in stromal fibroblasts had significantly poorer DFS as well as OS rates (Fig. 4b). In contrast, patients with moderate or high ATR expression levels showed better OS and DFS (*P* = 0.0001 and *P* = 0.0004, respectively) (Fig. 4b). Interestingly, patients with moderate levels of stromal ATR showed the best OS and DFS (Fig. 4b). Similarly, the ATR levels in cancer cells were correlated with patient survival (Fig. 4c). In these cases, patients with moderate and high ATR levels showed similar higher OS and DFS than patients with low ATR expression (*P* = 0.0006 and *P* = 0.0392, respectively) (Fig. 4c). Univariate Cox regression analysis also showed an increased risk for patients with low ATR levels in both stromal fibroblasts (hazard ratio > 1; *P* = 0.0001) and cancer cells (hazard ratio > 1; *P* = 0.0012) (Table 2). In order to establish whether the prognostic power of the ATR expression level is independent of other well-known breast cancer risk factors, multivariate Cox regression analysis was conducted. Table 3 indicates that the ATR expression level in stromal fibroblasts is a significant independent predictor of OS and DFS (*P* = 0.0019, *P* = 0.0285, respectively) (Table 3). Similarly, ATR expression in cancer cells was an independent prognostic factor for OS, but not for DFS (Table 3).

**Table 2:** Univariate Cox proportional regression analysis on 5-year overall and disease-free survival of 103 LABC patients

Parameter	Overall survival			Disease-free survival		
	Hazard ratio	95% Cl†	P*	Hazard ratio	95% Cl†	P*
**ATR (fibroblasts)**						
High	1.000			1.000		
Low	7.934	2.950–21.340	0.0001	0.406	0.198–0.833	0.0140
**ATR (epithelial cells)**						
High	1.000			1.000		
Low	7.711	2.244–26.498	0.0012	0.732	0.422–1.270	0.2670
**Tumor size**						
≤ 5 cm	1.000			1.000		
> 5 cm	1.239	0.439–3.500	0.6852	0.796	0.452–1.404	0.4311
**Age**						
≤ 52 years	1.000			1.000		
> 52 years	0.984	0.940–1.030	0.4826	1.012	0.985–1.039	0.3854
**Stage**						
T2	1.000			1.000		
T3, T4, Tx	1.941	0.642–5.866	0.2401	0.508	0.292–0.883	0.0163
**Grade**						
I/II	1.000			1.000		
Ш/poorly differentiated	1.949	0.758–5.012	0.1660	1.241	0.723–2.130	0.4328

**Table 3:** Multivariate Cox regression analysis on 5-year overall and disease-free survival

**Parameter**	**Overall survival**			**Disease-free survival**		
	**Hazard ratio**	**95% Cl†**	P*	**Hazard ratio**	**95% Cl†**	P*
**ATR (fibroblasts)**	14.708	2.693–80.332	0.0019	0.369	0.151–0.900	0.0285
**Tumor size**	0.665	0.084–5.244	0.6985	1.018	0.276–3.759	0.9790
**Age**	0.878	0.802–0.962	0.0051	1.027	0.989–1.067	0.1642
**Stage**	95.600	5.109–1788.693	0.0023	0.471	0.112–1.975	0.3035
**Grade**	0.868	0.187–4.030	0.8564	1.363	0.647–2.874	0.4155
**HR_ Status** ER(+ve)/Her2(−ve)	8.981	0.774–104.259	0.0793	0.649	0.283–1.489	0.3079
**HR_ Status** ER(−ve)/Her2(+ve)	31.436	2.109–468.544	0.0124	0.412	0.102–1.662	0.2096
**HR_ Status** ER(−ve)/Her2(−ve)	30.258	2.054–445.803	0.0130	0.592	0.161–2.231	0.4445
**ER_PR_Status** ER(+ve)/PR(−ve)	0.074	0.003.009–1.771	0.1080	0.759	0.110–5.254	0.7802
**ER_PR_Status** ER(+ve)/PR(+ve)	0.845	0.122–5.837	0.8640	0.726	0.169–3.108	0.6655
**Parameter**	**Overall survival**			**Disease-free survival**		
	**Hazard ratio**	**95% Cl†**	P*	**Hazard ratio**	**95% Cl†**	P*
**ATR (epithelial cells)**	10.242	1.745–60.111	0.0100	0.834	0.436–1.598	0.5849
**Tumor size**	0.049	0.006–0.425	0.0062	1.459	0.402–5.301	0.5659
**Age**	0.880	0.804–0.962	0.0050	1.028	0.989–1.068	0.163**7**
**Stage**	257.360	13.853–4781.270	0.0002	0.334	0.084–1.325	0.1188
**Grade**	1.205	0.266–5.452	0.8083	1.379	0.672–2.832	0.3807
**HR_ Status** ER(+ve)/Her2(−ve)	2.731	0.382–19.517	0.3166	0.633	0.266–1.505	0.3004
**HR_ Status** ER(−ve)/Her2(+ve)	1.456	0.146–14.486	0.7488	0.657	0.196–2.207	0.4973
**HR_ Status** ER(−ve)/Her2(−ve)	4.817	0.459–50.592	0.1901	0.655	0.193–2.225	0.4973
**ER_PR_Status** ER(+ve)/PR(−ve)	0.901	0.465–25.058	0.9296	1.187	0.207–6.820	0.8474
**ER_PR_Status** ER(+ve)/PR(+ve)	10.511	0.671–164.698	0.2275	1.021	0.983–1.061	0.2730

## Discussion

It has become clear that breast stromal fibroblasts play important roles in tumor spreading and relapse as well as the therapeutic outcome of patients. Furthermore, recent findings underscored CAFs as important potential diagnostic and/or prognostic tool in clinical practice [3, 18, 19]. Therefore, it is of utmost importance to further characterize these cells and determine relevant predictive markers. In the present study, we have shown that the cell cycle protein kinase ATR is lowly expressed in CAFs compared to their corresponding cells present in adjacent histologically normal tissues. This corroborates our previous results obtained in cultured CAF/TCF cells [8]. This differential expression suggests that ATR downregulation in CAFs is due to the presence of these fibroblasts close to cancer cells, which perturbs the microenvironment through paracrine signaling. In fact, we have recently shown that cancer cells activate breast stromal fibroblasts in an IL-6-dependent manner [20]. Therefore, we tested the effect of breast cancer cells and IL-6 on the expression of ATR in BSFs, and have shown that breast cancer cells as well as pure recombinant IL-6 downregulate ATR in BSFs. This effect was mediated through IL-6-dependent activation of STAT3 and its downstream target AUF1. This prompted us to elucidate the link between AUF1 and ATR and have shown that AUF1 destabilizes the *ATR* mRNA through binding its 3′UTR and accelerates its turnover, which explains ATR downregulation in active BSFs. This has been summarized in Fig. 2f. Similar AUF1-related post-transcriptional regulation has been previously shown for p16 in breast myofibroblasts [21]. AUF1 is also a repressor of p53 [22], which indicates that AUF1 upregulation is a major step toward BSF transactivation. Indeed, we have previously shown that AUF1 is upregulated in 66% CAFs relative to their adjacent TCF counterparts [21]. This confirms that ATR is another important target of breast cancer cells in BSFs, and that this protein kinase plays key roles in the carcinoma-stroma cross-talk during breast carcinogenesis. The procarcinogenic effects of ATR-deficient breast stromal fibroblasts were shown in vitro using direct coculturing in engineered human breast tissues composed of breast cancer cells and BSFs. Indeed, ATR-deficient cells enhanced the growth of breast cancer cells, which was confirmed by showing the expression of high levels of Ki-67 and cyclin D1. Similarly, we have recently shown that ATR downregulation activates BSFs and enhance their paracrine procarcinogenic effects both in vitro and ion orthotopic tumor xenografts [8]. These protumorigenic effects of ATR-deficient breast stromal fibroblasts prompted us to evaluate the consequences of ATR downregulation in cancer cells as well as their stromal fibroblasts on the survival of patients with LABC treated with neoadjuvant therapy, which is widely used for the treatment of these high-risk breast cancer patients. Therefore, identification of better markers of response is highly needed for a better stratification of patients. Importantly, we have shown significant association between ATR expression levels in both tumoral and stromal cells and patient’s overall survival as well as disease-free survival. In both cell types, absence or reduced ATR expression predicted poor clinical outcome in locally advanced breast cancers. This indicates that low ATR levels in cancer and/or stromal fibroblasts in these tumors can significantly predict high risk of recurrence. Interestingly, while moderate and high ATR levels in cancer cells were similarly correlated with better outcome, moderate ATR levels in stromal fibroblasts were correlated with better patient outcome than those who expressed high ATR levels (Fig. 4). Furthermore, ATR expression in CAFs was inversely correlated with tumor recurrence, progression, and patient survival, which was also highly correlated with the level of ATR in breast cancer cells.

Univariate Cox regression analysis confirmed that low ATR levels in both stromal fibroblasts and cancer cells were a significant indicator of poor clinical outcome. Moreover, multivariate Cox regression analysis showed that the ATR expression level in stromal fibroblasts is a significant independent predictor of OS and DFS, while ATR level in cancer cells was an independent prognostic factor only for OS, but not for DFS (Table 3). This indicates that the ATR expression level in both cancer as well as stromal fibroblasts could be of great prognostic value for patients with LABC treated with neoadjuvant therapy.

## Conclusions

The present findings show that ATR is downregulated in cancer cells and their neighboring CAFs in breast cancer tissues as compared to their respective adjacent normal tissues. Furthermore, we present clear indication that low levels of ATR in tumor cells as well as their adjacent stromal fibroblasts predict high risk of recurrence and poor survival post-neoadjuvant treatment of locally advanced breast cancer patients. Therefore, the level of this protein kinase in breast cancer cells or in the adjacent stromal fibroblasts could constitute a powerful prognostic biomarker for these hard-to-treat patients who need downstaging tumors to facilitate breast conservation therapy.

## Supplementary information

**Additional file 1.** Clinicopathological characteristics of the breast cancer patients. This table summarizes the clinicopathological features of the patients.

**Additional file 2.** Expression of ATR in cancer cells and stromal fibroblasts. This table is showing the expression of ATR in four different breast cancer sub-types.

## Data Availability

The data generated, used, and analyzed in the current study are available from the corresponding author in response to a reasonable request.

## Abbreviations

ATR: *Ataxia telangiectasia* and Rad3-related
BSF: Breast stromal fibroblast
CAF: Cancer-associated fibroblast
DFS: Disease-free survival
IL-6: Interleukin-6
LABC: Locally advanced breast cancer
OS: Overall survival
SFM: Serum-free media
SFCM: Serum-free conditioned media
TCF: Tumor counterpart fibroblast
