# Supplementary material for: Clinical and functional significance of tumor/stromal ATR expression in breast cancer patients
Source: Breast Cancer Res. 2020 May 15;22:49. doi: 10.1186/s13058-020-01289-4 (PMC7229635; doi:10.1186/s13058-020-01289-4)
Supplement: Supplementary file 1 — Additional file 1. Clinicopathological characteristics of the breast cancer patients. This table summarizes the clinicopathological features of the patients. [file 13058_2020_1289_MOESM1_ESM.docx]

Additional file 1. Clinicopathological characteristics of the breast cancer patients

| **Parameter** | **Total (n= 103) (%)** | **ER (+ve)/Her2 (+ve)** | **ER (+ve)/Her2 (-ve)** | **ER (-ve)/Her2 (+ve)** | **ER (-ve)/Her2 (-ve)** | **P value** |
| --- | --- | --- | --- | --- | --- | --- |
| **Age (years** |  |  |  |  |  |  |
| ≤50 | 72 (69.90) | 19 (18.45) | 19 (18.45) | 18 (17.48) | 16 (15.53) | 0.4751 |
| ˃50 | 31 (30.10) | 7 (6.80) | 13 (12.62) | 6 (5.83) | 5 (4.85) |  |
| **Stage** |  |  |  |  |  |  |
| T2 | 30 (29.13) | 3 (2.91) | 8 (7.77) | 10 (9.71) | 9 (8.74) | 0.1147 |
| T3 | 29 (28.16) | 11 (10.68) | 10 (9.71) | 3 (2.91) | 5 (4.85) |  |
| T4 | 43 (41.75) | 12 (11.65) | 13 (12.62) | 11 (10.86) | 7 (6.80) |  |
| Tx | 1 (0.97) | 0 (0.00) | 1 (0.97) | 0 (0.00) | 0 (0.00) |  |
| **KI-67 index (%)** |  |  |  |  |  |  |
| 0 | 85 (84.16) | 22 | 23 | 21 | 19 (18.81) | 0.2417 |
| ≤15 | 2 (1.98) | 0 (0.00) | 2 (1.98) | 0 (0.00) | 0 (0.00) |  |
| ˃15 | 14 (13.86) | 3 (2.97) | 7 (6.93) | 3 (2.97) | 1 (0.99) |  |
| **Histological subtypes** |  |  |  |  |  |  |
| **None Invasive** | 1 (0.97) | 0 (0.00) | 0 (0.00) | 1 (0.97) | 0 (0.00) |  |
| **Invasive Ductal Ca** | 85 (82.52) | 21 (20.39) | 25 (24.27) | 19 (18.45) | 20 (19.42) |  |
| **1,4** | 3 (2.91) | 0 (0.00) | 2 (1.94) | 1 (0.97) | 0 (0.00) | 0.6416 |
| **Invasive Ductal Ca with DCIS** | 11 (10.68) | 4 (3.88) | 3 (2.91) | 3 (2.91) | 1 (0.97) |  |
| **Infiltrating Lobular Ca** | 1 (0.97) | 0 (0.00) | 1 (0.97) | 0 (0.00) | 0 (0.00) |  |
| **3, 4** | 1 (0.97) | 0 (0.00) | 1 (0.97) | 0 (0.00) | 0 (0.00) |  |
| **Other** | 1 (0.97) | 1 (0.97) | 0 (0.00) | 0 (0.00) | 0 (0.00) |  |
| **Recurrence** |  |  |  |  |  |  |
| **No** | 63 (61.76) | 21 (20.59) | 21 (20.59) | 12 (11.76) | 9 (8.82) | 0.044 |
| **Yes** | 39 (38.24) | 5 (4.90) | 11 (10.78) | 12 (11.76) | 11 (10.78) |  |
| **Grade** |  |  |  |  |  |  |
| **G1** | 3 (2.91) | 0 (0.00) | 3 (2.91) | 0 (0.00) | 0 (0.00) |  |
| **G2** | 46 (44.66) | 10 (9.71) | 20 (19.42) | 10 (9.71) | 6 (5.83) | 0.0136 |
| **G3** | 54 (52.43) | 16 (15.53) | 9 (8.74) | 14 (13.59) | 15 (14.56) |  |
| **Tumor size** |  |  |  |  |  |  |
| **≤5** | 35 (40.23) | 4 (4.60) | 10 (11.49) | 11 (12.64) | 10 (11.49) | 0.0305 |
| **˃5** | 52 (59.77) | 18 (20.69) | 18 (20.69) | 8 (9.20) | 8 (9.20) |  |
| **Progression** |  |  |  |  |  |  |
| **No** | 73 (72.28) | 21 (20.79) | 23 (22.77) | 18 (17.82) | 11 (10.89) | 0.2550 |
| **Yes** | 28 (27.72) | 5 (4.95) | 8 (7.92) | 6 (5.94) | 9 (8.91) |  |
| **Survival Status** |  |  |  |  |  |  |
| **Alive** | 84 (81.55) | 22 (21.36) | 29 (28.16) | 19 (18.45) | 14 (13.59) | 0.1648 |
| **Dead** | 19 (18.45) | 4 (3.88) | 3 (2.91) | 5 (4.85) | 7 (6.80) |  |
| **Duration of clinical follow-up (Years, mean±SD)** |  | 4.81307692 | 4.91031250 | 3.98333333 | 3.85285714 | 0.1251 |
