# Supplementary material for: Clinical and functional significance of tumor/stromal ATR expression in breast cancer patients
Source: Breast Cancer Res. 2020 May 15;22:49. doi: 10.1186/s13058-020-01289-4 (PMC7229635; doi:10.1186/s13058-020-01289-4)
Supplement: Supplementary file 2 — Additional file 2. Expression of ATR in cancer cells and stromal fibroblasts. This table is showing the expression of ATR in four different breast cancer sub-types. [file 13058_2020_1289_MOESM2_ESM.docx]

**Additional file 2**. Expression of ATR in cancer cells and stromal fibroblasts

| **Epithelial cells** | **Total (n= 103) (%)** | **ER (+ve)/Her2 (+ve)** | **ER (+ve)/Her2 (-ve)** | **ER (-ve)/Her2 (+ve)** | **ER (-ve)/Her2 (-ve)** | **P value** |
| --- | --- | --- | --- | --- | --- | --- |
| **ATR fibroblasts** |  |  |  |  |  |  |
| High | 27 (26.21) | 7 (6.80) | 6 (5.83) | 5 (4.85) | 9 (8.74) |  |
| Intermediate | 30 (29.13) | 12 (11.65) | 7 (6.80) | 4 (3.88) | 7 (6.80) | 0.0212 |
| low | 46 (44.66) | 12 (11.65) | 19 (18.45) | 15 (14.56) | 5 (4.85) |  |
| **ATR Epithelial** |  |  |  |  |  |  |
| High | 46 (44.66) | 9 (8.74) | 13 (12.62) | 12 (11.65) | 12 (11.65) |  |
| Intermediate | 19 (18.45) | 7 (6.80) | 8 (7.77) | 3 (2.91) | 1 (0.97) | 0.461 |
| low | 38 (36.89) | 10 (9.71) | 11 (10.68) | 9 (8.74) | 8 (7.77) |  |
